# Cell identity specification in plants: lessons from flower development

**DOI:** 10.1093/jxb/erab110

**Published:** 2021-04-17

**Authors:** Xiaocai Xu, Cezary Smaczniak, Jose M Muino, Kerstin Kaufmann

**Affiliations:** 1 Plant Cell and Molecular Biology, Institute of Biology, Humboldt-Universität zu Berlin, Berlin, Germany; 2 Systems Biology of Gene Regulation, Humboldt-Universität zu Berlin, Institute of Biology, Berlin, Germany; 3 University College Dublin, Ireland

**Keywords:** Cell lineage, cellular differentiation, flower development, phytohormone, plant cell type, positional regulation, transcription factor

## Abstract

Multicellular organisms display a fascinating complexity of cellular identities and patterns of diversification. The concept of ‘cell type’ aims to describe and categorize this complexity. In this review, we discuss the traditional concept of cell types and highlight the impact of single-cell technologies and spatial omics on the understanding of cellular differentiation in plants. We summarize and compare position-based and lineage-based mechanisms of cell identity specification using flower development as a model system. More than understanding ontogenetic origins of differentiated cells, an important question in plant science is to understand their position- and developmental stage-specific heterogeneity. Combinatorial action and crosstalk of external and internal signals is the key to cellular heterogeneity, often converging on transcription factors that orchestrate gene expression programs.

## Teaser

Cellular differentiation in plants integrates information from developmental history, position, and environment. We use flower development as a model system to introduce molecular mechanisms controlling cell type specification and cellular heterogeneity.

## Introduction

### How to define a cell type in plants

#### The cell type concept

A cell type is classically defined by its phenotype and function. More recently, molecular signatures such as gene expression profiles and epigenetic patterns have been introduced to assist in defining and distinguishing cell types (Brady *et al.*, 2007; Yadav *et al.*, 2014; X. Zhang *et al.*, 2018). Less than 20 major cell types are classically assigned in vascular plants (Steeves and Sussex, 1989). Thus classical cell type concepts aim to generalize cellular identity and may not entirely cover the complexity and cellular diversity of cells within the major tissue and organ types in plants.

In 1665, Robert Hooke first discovered the cells in a piece of cork tissue, calling them ‘pores’ and later naming those structures ‘cells’, implying both the form and function of the cells (Mazzarello, 1999). The botanist Matthias Jakob Schleiden (1804–1881) later suggested that every structural element of plants is composed of cells or their products (Schleiden, 1838). With the improvement of microscopy and anatomy, cell types have historically been defined by morphology, localization, ontogeny, and function (e.g. Carter *et al.*, 1986). Concepts of plant development have traditionally been strongly entangled with the question of ontogenetic (and later evolutionary) origins of morphological structures. Classical concepts aim to trace the origins of ‘novel’ organs or structures from pre-existing structures, for example by exploring and conceptualizing ‘hidden’ evolutionary relationships and commonalities of shoots, and vegetative and floral organs (see, for example, Arber, 1950). With the origins of molecular genetics, evolutionary developmental biology has revolutionized our understanding of the molecular mechanisms underlying the evolutionary origins and diversification of plant organs and cell types. Gene duplications followed by sub- and neofunctionalization, co-option of genes, and gene regulatory modules were found to contribute to the striking morphological complexity in higher plant species. However, in very practical terms, this creates challenges, for instance since many developmental control genes act in more than one developmental process linked to evolutionary history. For example, many genes controlling vegetative leaf development are also expressed in floral organs, and their activities are modulated to give rise to specific structures of floral organs. This often makes it hard to identify marker genes that are very specifically expressed in only one cell type or differentiation stage. One might compare the problem of defining a ‘cell type’ in plants with that of the ‘species concepts’ in plants (Christenhusz, 2020), in that it can be difficult to distinguish cell ‘types’ from ‘states’, and cells with the same ‘identity’ may appear very different from each other in terms of gene expression profiles. The challenge is thus to deduce cell ‘history’, similar to deducing the natural history of species.

**Fig. 1.**
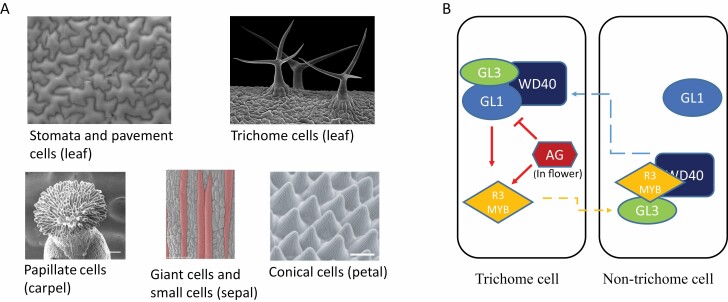
Diversity of epidermal cell types and control of trichome development. (A) Epidermal cell types, including stomatal guard cells and pavement cells on a leaf, trichome cells on a leaf, papillate cells on a carpel (scale bar, 500 μm), giant cells and small cells on a sepal (scale bar, 100 μm), and conical cells on a petal (scale bar, 15 μm). (B) Movement of R3 MYB functions as a positional signal for trichome patterning (after Grebe, 2012). AG serves as an upstream lineage signal in flowers to suppress trichome formation. Solid lines represent direct regulation of target genes, and dashed lines represent movement. SEM images in (A) are reprinted with permission from Riglet *et al.* (2020) (‘papillate cells’), Meyer *et al.* (2017) (‘giant cells and small cells’), Yang *et al.* (2019) (‘conical cells’), Emmanuel Boutet (Plant biocurator for UniProtKB/Swiss-Prot) (‘stomata and pavement cells’), and Stefan Eberhard (University of Georgia, USA; provided by Wellcome collection under a CC BY-NC 4.0 licence) (‘trichomes’).

The idea that a generalized cell type concept does not fully reflect the cellular diversity in form and function in plants can be illustrated by the cell types that together constitute the epidermis tissue (Fig. 1A). The plant epidermis forms the outer cell layer of the plant like the skin of the human body, and its primary functions are to protect inner tissues and to act as the communication and exchange surface with the environment (Glover, 2000). Epidermal pavement cells of Arabidopsis leaves are usually shaped like the interlocking pieces of a jigsaw puzzle. Pavement cells in sepals instead are boxy and differentiated into giant and small cells, while the adaxial epidermal cells in petals are conical and uniform (Glover, 2000; Roeder *et al.*, 2010; Huang and Irish, 2015). Meristemoids within the abaxial epidermis of developing leaves and in floral organs give rise to stomata. Trichomes develop typically at regular distances in the abaxial side of leaves and sepals; however, trichome anatomy is highly variable among organs of a plant. Cellular characteristics reflect functional differences of epidermis cells among organs. For example, epidermal cells in petals enhance attractiveness to pollinators. Specialized epidermal cells in carpels, the stigma, receive and induce the germination of pollen grains (Glover and Martin, 1998; Balanzà *et al.*, 2014). Together, the example of the epidermis shows that the cell type composition of tissues can be variable across organs in a plant, and that individual cell types can have different phenotypes and function. Even more, cellular morphology and cell type frequencies within tissues can be plastic and affected by environmental factors (see, for example, Wardlaw, 1952; Casson and Gray, 2008).

Cellular differentiation in plants can be reversible under specific conditions. Plants are sessile organisms and have to cope with injuries caused by environmental stimuli or biotic attacks. Somatic cells can be reprogrammed to regenerate new organs or repair damaged tissues (Ikeuchi *et al.*, 2019). Laser-assisted elimination of cells in Arabidopsis root triggers the cells adjacent to the injury to re-activate stem cell pathways, change the cell division orientation accordingly, and acquire the cell fates of the missing cells to replace them (Marhava *et al.*, 2019). Other kinds of stresses, such as osmotic, heavy metal ion, and dehydration stress, can also induce plant cells to regenerate (Ikeda-Iwai *et al.*, 2003). The questions remain of which factors and mechanisms determine stem cell identity in plants, how different stem cell niches can be distinguished from one another (beyond the activity of some master regulators), and what are the exact mechanisms underlying dedifferentiation versus terminal differentiation of specific cell types.

Altogether, the concept of ‘cell type’ aims to simplify the complex nature of cellular diversity in multicellular plants (and animals). Therefore, it is important to understand the origins and consequences of heterogeneity within and among cell types, and the mechanisms underlying their organ- and environment-specific modulation.

#### Cell lineage versus positional signals

Understanding the principles that govern cell type specification in multicellular organisms is one of the major challenges in developmental biology. The fundamental concept of ‘epigenetic landscape’ introduced by Waddington in 1957 visualizes cell differentiation as a ball rolling down a valley in a landscape that is sculpted by regulatory genes and their combinatorial activities (Waddington, 1957). This path forms the developmental trajectory or ‘lineage’ of the cell defined by its start position and by dynamic but predictable changes in gene activities determined by the regulators. In the animal field, reconstruction of cell lineage history is often used to understand cellular differentiation programs (Kretzschmar and Watt, 2012; Morris, 2019). A classical example for utilizing the lineage concept in plants is stomata development, which is initiated from a lineage-specific stem cell via a series of defined asymmetric cell divisions controlled by consecutively acting regulatory factors and enforced by cell–cell signaling (Han and Torii, 2016).

However, many experiments have shown that the developmental fate of a plant cell does not depend strictly on its lineage, but on its exact position within the growing plant body (Stewart and Dermen, 1975; van den Berg *et al.*, 1995; Szymkowiak and Sussex, 1996; Berger *et al.*, 1998; Kidner *et al.*, 2000; Costa and Shaw, 2006; Costa, 2016). In early studies, plant scientists utilized mosaic or chimera experiments to trace the lineage of a cell type by labeling cell clones with genetically phenotypic traits, such as ploidy level or albinism, and tracking the mitotic descendants of marked cells (Poethig, 1987, 1989; Dawe and Freeling, 1991; Irish, 1991; Scheres, 2001). Chimera studies have shown that plant cells do not follow strict lineages, and their fates are not pre-determined but rely on positional information or cell–cell interactions (Szymkowiak and Sussex, 1996). For instance, during leaf development, epidermal cells regularly undergo anticlinal divisions to form the outermost layer. However, in chimeras, occasionally small sectors derived from periclinal divisions of epidermal cells have been observed. These cells, although of epidermis lineage, adapt to their new position and differentiate as internal mesophyll cells (Stewart and Burk, 1970; Stewart and Dermen, 1975). This phenomenon has also been observed in the root meristem. If a cortical initial cell is laser ablated, the adjacent pericycle cell switches its fate and takes the position of the cortical cell to continue forming corresponding cell files (van den Berg *et al.*, 1995). Plant cells may alter their identity when positional signals are changed. This is exemplified by observations on wound healing and de-/re-differentiation. In the past ~30 years, the molecular nature of many signaling mechanisms controlling cellular differentiation and cell type specification in plants has been elucidated. Combinations of genetic analyses and computational modeling have allowed us to gain insights into the regular nature of the positioning mechanisms.

## Signals controlling cell type specification in plants

Different mechanisms explaining the relationships between relative position, spatial patterns, and cell fate in developing organisms have been proposed. A major and often considered mechanism is the formation of gradients of signaling molecules, such as morphogens, that result in the specification of distinct cell fates in a concentration-dependent manner (Wolpert, 1996). Mobile signaling molecules in plants reported to control patterning include phytohormones, mobile transcription factors (TFs), non-coding RNAs, and small signaling peptides. A second general model for explaining position-dependent cell specification does not rely on a gradient, but on biochemical signaling between neighboring cells in ‘boundary’ regions, essentially resulting in self-organization of the system (Sharpe, 2019). The idea that mechanical signals play a role in cellular differentiation and patterning in plants has been recognized for a long time (see, for example, Arber, 1950), but is also an exciting focus of ongoing research.

### The phytohormone auxin in flower development

Graded auxin accumulation has been shown to play important roles in developmental patterning, while a primary effect is on cell expansion (Leyser, 2018), thereby controlling processes such as vascular development and specification of floral meristem founder cells. Auxin is actively transported in a polar manner between cells via transport proteins, such as PIN-FORMED (PIN) efflux carriers. The *pin1* mutant fails to produce flowers and presents a pin-shaped inflorescence (Okada *et al.*, 1991). Flower initiation can be rescued when indole acetic acid (IAA) is applied exogenously to the *pin1* mutant (Reinhardt *et al.*, 2000). Cellular specificity of auxin responses is linked to the complexity of auxin sensing and response pathways in the cell (Leyser, 2018). After binding to receptors of the TRANSPORT INHIBITOR RESPONSE1/AUXIN SIGNALING FBOX (TIR1/AFB) family, auxin controls gene expression in the nucleus via activation of AUXIN RESPONSE FACTOR (ARF) TFs by targeted degradation of AUXIN/INDOLE-3ACETIC ACID (AUX/IAA) repressor proteins (Weijers and Wagner, 2016). The initiation of flower meristems is marked by the formation of local maxima of auxin (Benková *et al.*, 2003; Heisler *et al.*, 2005). A series of auxin responses is started by the activation of ARF5 (also known as MONOPTEROS), which promotes the expression of TFs mediating floral meristem identity, including LEAFY (LFY) (Yamaguchi *et al.*, 2013, 2016). LFY further promotes the expression of *APETALA1* (*AP1*) (Parcy *et al.*, 1998; Wagner *et al.*, 1999). LFY and AP1 play a central role in flower meristem specification and they regulate a large number of downstream genes for flower formation (Parcy *et al.*, 1998; Kaufmann *et al.*, 2010b; Winter *et al.*, 2011). Besides a role in early activation of flower development, auxin has an instructive role in patterning of organs within the flower, as revealed by higher order *yucca* mutants that are defective in auxin biosynthesis (Zhao *et al.*, 2001; Tobeña-Santamaria *et al.*, 2002; Cheng *et al.*, 2006). Besides this, other ARF TFs were found to direct organ polarity as well as stamen differentiation (Przemeck *et al.*, 1996; Sessions *et al.*, 1997; Nagpal *et al.*, 2005; Simonini *et al.*, 2016; K. Zhang *et al.*, 2018). To explain the multiple functions of auxin, we need a better understanding of the cell type-specific composition of the auxin response machineries. This requires knowledge of quantitative abundance of specific auxin signaling factors across cellular differentiation in the flower, direct ARF targets, along with their affinity and specificity for protein–protein and protein–DNA interactions. At the same time, the chromatin landscape of a cell type may affect auxin response. Addressing these questions will help to understand how transient peaks in auxin concentration can trigger cellular patterning responses, and to distinguish direct effects from indirect downstream developmental decisions.

### Mobile regulatory molecules in flower development

Small peptides are usually <20 amino acids long in the mature form and rarely longer than 120 amino acids as a precursor, and they are often present in very low physiological concentrations in the nanomolar range (Murphy *et al.*, 2012). Intercellular communications via mobile peptides and receptor signaling cascades have been identified as important regulators of cell identity in meristems and during organ tissue differentiation (see, for example, Lee *et al.*, 2015; Lin *et al.*, 2017). A classical example for this is the CLE (CLV3/ESR3, EMBRYONIC SURROUNDING REGION) family which controls stem cell identity in the meristems of the plant. By this, the CLE family impacts flower development, since the size of the stem cell niche in the floral meristem has a direct effect on the number of floral organs that are produced by it. The *clv3* mutant leads to overproliferation of the shoot apical meristem (SAM) cell population, while overexpression of CLV3 eliminates the stem cell niche and results in termination of shoot development (Fletcher *et al.*, 1999; Kondo *et al.*, 2006). *CLV3* is shown to be expressed in stem cells at the meristem apex. It diffuses toward inner layers of the meristem and organizing center, where it interacts with the leucine-rich receptor-like kinase (LRR‐RLK) CLV1 and related proteins to restrict the expression of a homeodomain TF protein WUSCHEL (WUS), which is a positive regulator of the stem cell population (Mayer *et al.*, 1998; Fletcher *et al.*, 1999; Schoof *et al.*, 2000; Kinoshita *et al.*, 2010). Furthermore, WUS can also migrate toward the *CLV3*-expressing cell layers, where it activates *CLV3* expression by directly binding to its genomic region (Yadav *et al.*, 2011). This negative feedback loop between WUS and CLV3 is well established to maintain a proper cell population in the SAM. Consequently, this pathway regulates meristem size and by this controls the number of organs produced in a flower: *clv3* mutants typically produce more floral organs, while overexpression results in flowers without inner whorls of stamens or carpels (Fletcher *et al.*, 1999).

miRNAs function by binding to complementary sites in mRNA molecules to trigger the degradation or translational inhibition of target genes. Small RNAs can diffuse across tissues, resulting in concentration gradients, thereby potentially mediating tissue patterning through dose-dependent activity (D’Ario *et al.*, 2017). miRNAs have been found to regulate the activity of TFs and other regulatory proteins in flower development. A classical example is miR172, which accumulates in the SAM at the onset of flowering, preventing the transition of the center of the SAM into a flower meristem by inhibiting *AP2* expression (Aukerman and Sakai, 2003; Chen, 2004). miR172 also accumulates in the center of the flower primordia to suppress *AP2*, avoiding *AP2* and *AG* co-expression and thereby setting the boundary between petal and stamen whorls. *miR172* is itself down-regulated by AP2, establishing a negative feedback loop that is essential for the correct specification of organ identity (Zhao *et al.*, 2007; Wollmann *et al.*, 2010). Several miRNAs are involved in the process of floral organ development partially by crosstalking with plant hormones. For example, miR167 directly targets the transcripts of *AUXIN RESPONSE FACTOR 6* (*ARF6*) and *AUXIN RESPONSE FACTOR 8* (*ARF8*) to regulate stamen filament and pollen development (Wu *et al.*, 2006). Furthermore, miR393 has been shown to target TIR1/AFB proteins, which are critical components of auxin signaling transduction (Parry *et al.*, 2009).

Concentration gradients formed by mobile TFs also play roles in developmental patterning (Vadde *et al.*, 2020). For instance, trichomes are equally distributed on leaves, because they can inhibit neighboring cells from acquiring trichome identity, and this program is modified in floral organs. In trichome-forming cells, the TF GLABRA 3 (GL3) forms a complex with GL1, activating not only the expression of positive regulators for trichome cell fate determination but also the expression of R3-MYB TFs. R3-MYB proteins form a complex with GL3 in the neighboring cells, thus preventing the formation of the GL3–GL1 complex (Pattanaik *et al.*, 2014). Cell fate decision during root hair development shares a similar mechanism, since movement of similar regulatory proteins between root hair and non-root hair cells reinforces their identity (Salazar-Henao *et al.*, 2016) (Fig. 1B). During flower development, trichome formation in carpels is suppressed by the floral homeotic AGAMOUS (AG) TF via several target pathways, including direct repression of *GL1* (Ó’Maoiléidigh *et al.*, 2018).

Together, these examples show that cell to cell signaling impacts developmental patterning and cell identity specification. Developmental programs thus represent the sum of ‘endogenous’ cellular status and different signaling cascades that emerge from cell to cell signaling.

### Mechanical signals in floral organ differentiation

The role of mechanical forces in developmental patterning has long been acknowledged. For example, in *The natural philosophy of plant form* (1950), Agnes Arber states ‘Judging merely from inspection, it looks as if limitation of the space into which to expand, and the actual pressure which the developing parts exert upon one another, must be the efficient cause [for different shapes of various members of the plant body]’.

Plant cells sense internal and external forces that can be perceived as growth signals that have important effects on and affect the shape of cells and organs (Hervieux *et al.*, 2017; Sapala *et al.*, 2018). Mechanical signals defining epidermal cell morphology are at least in part perceived by stress-dependent, katanin-mediated alignment of microtubules in the cytoplasm that in turn guide cellulose-synthesizing complexes in the apoplast (Hamant *et al.*, 2008; Jacques *et al.*, 2013; Sampathkumar *et al.*, 2014). Sepal development has been used as a model to study the contribution of mechanical signals to local growth because of its high variability in growth rate within tissues and its robustness in the final shape of the organ (Tauriello *et al.*, 2015; Hervieux *et al.*, 2017). Trichome precursor cells in sepals initially grow and expand much faster than surrounding epidermal cells, which potentially can distort the final shape of the sepal. However, neighboring cells of trichome precursors organize their microtubule arrays according to the mechanical changes caused by trichome precursor cells, and thus grow at a reduced rate to maintain sepal shape (Hervieux *et al.*, 2017). Mechanical forces are involved in the control of sepal size. Tangential tension at the tip of the sepal causes the arrest of growth at the tip by reorientation of the microtubule array (Hervieux *et al.*, 2016).

Another example of mechanical signaling comes from the SAM, where cells orient their cortical microtubules along the lines of mechanical stress generated during tissue formation, and this then affects the mechanical properties of the cell, thus establishing a feedback loop (Uyttewaal *et al.*, 2012). Mechanical forces crosstalk with biochemical signals, for instance in the generation of phyllotactic patterns. Auxin minima reside in organ boundaries, and these regions are characterized by the expression of a specific group of TFs, such as *CUP-SHAPED COTYLEDON1* (*CUC1*), *CUC2*, and *CUC3*, which limit cell growth and thus create a creased shape in the boundaries (Heisler *et al.*, 2005; Rast and Simon, 2008). Microtubule arrangement and polar auxin transport in the boundaries are regulated by mechanical forces (Heisler *et al.*, 2010; Landrein *et al.*, 2015). Mechanical signals interplay not only with auxin, but also with miRNA regulation. The expression of CUC genes in organ boundaries is regulated by both miRNA and mechanical forces (Fal *et al.*, 2016). Interestingly, it was reported that the shape of nuclei correlates with cell shape and size in plants (Meier *et al.*, 2016). Mechanotransduction can affect the shape of the nucleus via interaction with the cytoplasmic microtubule cytoskeleton, and ‘nuclear stiffness’ may affect transcriptional regulation and gene activity (Finan and Guilak, 2010; Goswami *et al.*, 2020b; Irianto *et al.*, 2013; Lovett *et al.*, 2013).

In summary, mechanical forces provide positional signals by regulating organ growth rate, cell and organ shape, thereby contributing to cellular differentiation and cell type frequencies within organs.

## Insights into lineage-based mechanisms

The cell lineage concept suggests that a cell’s fate is determined early by its progenitors. Cells pass on specific cell fate decisions to their progeny across cell division (Stent, 1985). Although positional mechanisms play an important role in cell fate specification in plants, lineage-based mechanisms cannot be neglected. If cell fate is specified and maintained solely by positional information, cells would have to re-establish their expression programs based on their new position at every division (Costa, 2016). However, data from roots suggest that cells that misexpress a cell identity marker gene frequently pass on the ‘wrong’ identity to their progeny (Costa, 2016). Once cell fate is specified by positional information, it is clonally maintained by lineage until they receive new positional input (Costa, 2016). The critical role of TFs and epigenetic regulators also indicates the existence of a lineage-based component in plant cell fate determination. Stomatal differentiation follows an evident cell lineage from meristemoid mother cells to mature guard cells (Yang and Sack, 1995; Larkin *et al.*, 1997; Nadeau and Sack, 2002). Several basic helix–loop–helix (bHLH) TFs—SPEECHLESS (SPCH), MUTE, and FAMA—function at each stage to control the lineage. The overexpression of SPCH induces extra asymmetric divisions and the production of excess stomata (MacAlister *et al.*, 2007). Ectopic MUTE expression in the petal that is normally devoid of stomata converted petal epidermal cells into stomata (Pillitteri *et al.*, 2008). Induced expression of FAMA transforms cotyledon epidermal cells into guard cells (Ohashi-Ito and Bergmann, 2006).

In more general terms, key TFs have been identified that trigger cell lineage differentiation, while the initial activation of these TFs may be dictated by positional signaling at early stages in plants and animals (Scott and Carroll, 1987; St Johnston and Nüsslein-Volhard, 1992; Scheres, 2001).

### Pioneer transcription factors, organ identity, and cell type specification

Over the past decades, genetic analyses have identified floral regulators and discovered detailed insights into how they interact and cooperate to control flower development. Plant morphogenesis depends on the combinatorial interplay of TFs to mediate distinct and dynamic spatiotemporal gene expression, associated with feedback control (Zik and Irish, 2003; Krizek and Fletcher, 2005; Alvarez-Buylla *et al.*, 2010; Ó’Maoiléidigh *et al.*, 2014; Thomson and Wellmer, 2019). Floral organ specification essentially requires modification of leaf developmental pathways, including changes in growth, cell type frequencies, cellular morphologies (e.g. trichomes in sepals versus leaves; conical cells in the petal epidermis) (Fig. 1A), and the establishment of flower-specific cell and tissue types that are not found in leaves (e.g. in reproductive organs).

Cell type specification requires orchestrated changes in global gene expression programs. So-called ‘pioneer TFs’ control cell type programming and reprogramming by promoting chromatin opening to make it accessible for other TFs (Zaret and Carroll, 2011; Iwafuchi-Doi and Zaret, 2014, 2016; Zaret and Mango, 2016; Lai *et al.*, 2018). Growing evidence suggests that pioneer TFs contribute to the regulation of developmental switches in plants (see, for example, Tao *et al.*, 2017, 2019; Lai *et al.*, 2018).

Several key TFs are required for the leaf-to-flower transition. LFY specifies flower meristem identity. Combinatorial expression of LFY and WUS induces the generation of floral organs on primary and lateral root tips (Gallois *et al.*, 2004), and inducible expression of LFY in root explants is sufficient to trigger flower formation, bypassing elaboration of a shoot (Weigel *et al.*, 1992; Wagner *et al.*, 2004). Furthermore, expression of the LFY co-regulator UNUSUAL FLORAL ORGANS (UFO) fused to a VP16 activation domain resulted in ectopic formation of flowers and inflorescences in vegetative leaves in the presence of a functional *LFY* gene (Risseeuw *et al.*, 2013).

Protein oligomerization via a SAM domain enabled LFY to bind to closed chromatin regions (Sayou *et al.*, 2016). The functions of the floral meristem factors LFY and AP1 are closely linked, and they regulate each other’s expression. For example, the expression level of *LFY* is reduced in *ap1 cal* double mutants, and the onset of expression of *AP1* is delayed in the *lfy* mutant (Weigel and Nilsson, 1995). The *ap1* mutant can suppress the terminal flower phenotype of the constitutive expression of *LFY* (Weigel and Nilsson, 1995). This cross-regulation is mediated by direct promoter interactions (Kaufmann *et al.*, 2010b; Winter *et al.*, 2011). Recent work also explains how LFY binds DNA in a nucleosomal context and enhances chromatin accessibility at its target loci such as *AP1* (Jin *et al.*, 2021; Lai *et al.*, 2021). The activation of *LFY* and *AP1* is furthermore under positional control by ARF5 (Wagner *et al.*, 1999; Yamaguchi *et al.*, 2013, 2016).

Floral organ identity is specified by homeotic TFs of the MADS-box family that interact in a combinatorial manner to specify different types of floral organs (Causier *et al.*, 2010). Combined loss of function of floral homeotic proteins results in the transformation of all floral organs into cauline leaf-like organs (Bowman *et al.*, 1991). Furthermore, the redundantly acting SEPALLATA (SEP) TFs are essential for the specification of all floral organ types. Accordingly, loss of SEP function results in the conversion of the floral organs into leaf-like structures (Pelaz *et al.*, 2000). SEP proteins act as mediators of higher order complex formation of other homeotic TF classes (Theißen and Saedler, 2001; Immink *et al.*, 2009). According to the floral quartet model, petals are specified by a complex consisting of AP1, APETALA3 (AP3), PISTILLATA (PI), and SEP proteins. In contrast, stamens are specified by a complex of AP3, PI, SEP, and AG proteins. Sepals are specified by complexes formed by SEP and AP1 proteins, while carpels are specified by a SEP/AG tetramer (Honma and Goto, 2001; Theissen, 2001).

Cooperative and combinatorial interactions are important for floral homeotic TFs and may facilitate their action as pioneer factors (Kaufmann and Airoldi, 2018). Vegetative leaves of transgenic plants that constitutively express SEP3–AP3–PI or AP1–AP3–PI are converted into petals, showing that these TFs are not only required but also sufficient to specify floral organ identity (Honma and Goto, 2001). SEM revealed that cells on both the abaxial and adaxial surface of the converted rosette leaves closely resembled cells on the surface of the petals (Pelaz *et al.*, 2001). Cauline leaves of AP3–PI–SEP3–AG ectopic expression lines were converted into staminoid organs, and all floral organs are transformed into stamens or staminoid organs. Homeotically converted cauline leaves of these transgenic plants consist of two distinct regions whose epidermal cells exhibit a morphology similarity to that of anthers and filaments, respectively (Honma and Goto, 2001). Combination of genome-wide ChIP-seq and Dnase I-seq time-series experiments suggested that AP1 and SEP3 facilitate the opening of closed chromatin and promote gene activation in flower development (Pajoro *et al.*, 2014), indicating roles as pioneer factors. Homeotic TFs are expressed throughout flower development. The analysis of target networks of homeotic TFs allows us to interrogate how homeotic TFs modulate organ growth and cellular morphology, and establish novel cell identities not found in vegetative leaves (Yan *et al.*, 2016; Chen *et al.*, 2018). For example, the homeotic gene *AG* acts in concert with the general organ polarity gene *KANADI1* to suppress trichome initiation in the carpel epidermis (Ó’Maoiléidigh *et al.*, 2018). An example for a flower-specific gene activation is *SPOROCYTELESS*, which is activated by AG in early stages of floral organ development and plays an essential role in patterning processes related to sporogenesis (Schiefthaler *et al.*, 1999; Yang *et al.*, 1999; Ito *et al.*, 2004). The finding that floral organs were derived from leaf-like organs during evolution can also explain the fact that many developmental TFs with roles in cellular patterning appear to act in more than one developmental process and display tissue- and organ specific functions, since this could be explained by evolutionry co-option and diversification of ancestral regulatory programs. In fact, this complicates analyses of cell identity in plants, since only a few genes are entirely characteristic to only one specific cell type. For example, TFs controlling abaxial and adaxial identity were recruited to control patterning in lateral organs, the stem and roots, and their activity can be modulated in a floral organ-specific manner (e.g. Siegfried *et al.*, 1999; Yamaguchi *et al.*, 2004).

### The role of epigenetics in plant cell lineage specification

While cell identity can be programmed by cell type-specific TFs, the robustness of the acquired transcriptional status depends on the chromatin environment (Hennig and Derkacheva, 2009; Costa and Dean, 2019). Epigenetic memory can be established during developmental progression because of stable and heritable epigenetic modifications (Huang *et al.*, 2013; Costa and Dean, 2019 and references therein). During cell division, the transcriptional status of genes can be recorded and transmitted to daughter cells via epigenetic regulation (Iwasaki and Paszkowski, 2014). To date, many epigenetic modifications have been found, such as DNA methylation, histone methylation, and histone acetylation. Chromatin modifications can be linked with gene activation or repression or a ‘poised’ state. For example, H3K4me3 and H3K36me3 are associated with gene activation, while H3K27me3 and H3K9me2 are commonly linked to transcriptional repression (Pikaard and Mittelsten Scheid, 2014).

How epigenetic modifications store and transmit ‘memory’ to daughter cells has been intensely studied. Inheritance of histone marks by daughter cells requires the collaboration between the DNA replication machinery, chromatin modifiers, and chromatin modifications themselves (Stewart-Morgan *et al.*, 2020). Polycomb factors are known factors controlling epigenetic memory across cell division that mediate trimethylation of histone 3 Lys27 (H3K27me3). During the DNA replication at mitosis, parental nucleosomes with H3K27 tri-methylation recruit polycomb repressive complex 2 (PRC2) which catalyzes the trimethylation on daughter strand DNA (Jiang and Berger, 2017). This mediates the stability of the repressed status at many developmental gene loci, across developmental stages, such as in the case of *FLOWERING LOCUS C* (*FLC*) (reviewed in Costa and Dean, 2019), and in a tissue-specific manner, such as in the case of *FLOWERING LOCUS T* (*FT*) (Farrona *et al.*, 2011). In more general terms, the phenotypes of Polycomb mutants strongly suggest broad roles in the mediation of developmental phase transitions and the commitment to cellular differentiation (Mozgova *et al.*, 2015).

Epigenetic marks at specific genomic loci can be dynamically regulated, for example by TFs that interact with epigenetic factors. Marks can be erased, re-written, or diluted by cell division. For example, the B3 domain TFs LEAFY COTYLEDON 2 (LEC2) and FUSCA3 (FUS3) displace VAL1 and VAL2 (two key components for Polycomb-mediated *FLC* silencing by vernalization) during early embryogenesis from the cold memory *cis*-element of *FLC* to disrupt Polycomb silencing and thus prevent H3K27me3 maintenance at *FLC* during the rapid embryonic cell divisions (Tao *et al.*, 2019). During flower initiation and morphogenesis, TFs such as LFY, MADS-box proteins, and ARF have been shown to modulate chromatin status by recruiting ATP-dependent nucleosome remodelers or general transcriptional co-regulators (Smaczniak *et al.*, 2012; Wu *et al.*, 2012, 2015). In sum, the current data suggest that epigenetic programming plays a role in cell lineage commitment in plants. The investigation of tissue- and stage-specific dynamics of epigenetic profiles can be expected to shed more light on the underlying molecular mechanisms.

### Synergistic action of position- and lineage-based cell fate control

**Fig. 2.**
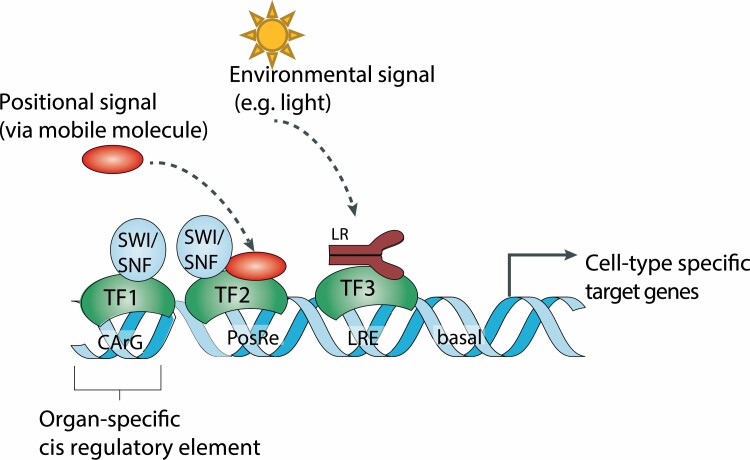
Cell type-specific gene expression integrates lineage, positional, and environmental gene expression. This model suggests an example mechanism of combinatorial control by TFs that act together, resulting in cell type-specific activation of target genes. Lineage (TF1)- and position (TF2)-specific factors can change the epigenetic status of the promoter region, for example by interacting with ATP-dependent nucleosome remodelers. In some instances, cell identity or anatomy can be modulated by environmental factors, thereby, for example, changing the frequency of cell types or cell shape in the epidermis. Abbreviations: LR, light receptor; SWI/SNF, SWItch/Sucrose Non-Fermentable (a type of ATP-dependent nucleosome remodeler); PosRe, position-responsive element; CArG. CArG-box (MADS TF-binding motif, as an example for organ-specific regulation); LRE, light-responsive element (an example for environmental regulation); basal, RNA polymerase II binding site.

The spatiotemporal expression pattern of floral homeotic TFs, and thereby the whorled organization of the flower, is facilitated by multiple factors, including epigenetic factors, positional signals, and regulatory feedback control (Alvarez-Buylla *et al.*, 2010; Denay *et al.*, 2017; Thomson and Wellmer, 2019). An example is provided by *AG* activity that is restricted to the inner whorls of the floral meristem giving rise to stamens and carpels. Besides being a PcG (Polycomb Group) target, *AG* expression is prevented in the outer floral whorls via the activity of histone deacetylases (Tian and Chen, 2001; Chen and Tian, 2007). Moreover, miR172 acts as a positional signal to restrict *AG* action by regulating the spatiotemporal activity of *AP2*, which is a known repressor of *AG* (Aukerman and Sakai, 2003; Chen, 2004; Zhao *et al.*, 2007; Wollmann *et al.*, 2010). Activation of AG in the inner whorls is mediated by the combined activity of several factors including WUS, LFY, and PERIANTHIA (Lenhard *et al.*, 2001; Lohmann *et al.*, 2001; Maier *et al.*, 2009). An autoregulatory feedback loop, possibly involving the interaction with SEP factors, contributes to the stable *AG* activity (Gomez-Mena *et al.*, 2005; Kaufmann *et al.*, 2009). This and other examples show that spatiotemporal gene expression determining cell identity requires combinatorial interplay of several factors (Fig. 2), and emphasizes the need for novel technological and computational approaches to understand the underlying *cis*-regulatory grammar.

Excellent examples for crosstalk of epigenetics and phytohormones have been described in controlling floral meristem specification. In general, some phytohormones, such as auxin, gibberellic acid, and brassinosteroids, have been shown to affect epigenetic modifications (Yamamuro *et al.*, 2016). In the absence of auxin, Aux/IAA proteins repress ARF5 activity in the SAM by interacting with ARF5 and recruiting the transcriptional co-repressor TOPLESS (TPL). TPL in turn interacts with histone deacetylase HDA19, thus removing histone acetylation at ARF5 target loci, thereby preventing gene activation (Eberharter and Becker, 2002; Long *et al.*, 2006; Szemenyei *et al.*, 2008). Upon auxin sensing, Aux/IAA proteins are rapidly degraded, leading to the dissociation of TPL and HDA19, thereby freeing ARF5 to activate its targets (Wu *et al.*, 2015; Lavy and Estelle, 2016). Furthermore, in the presence of auxin, ARF5 recruits ATP-dependent SWI/SNF remodeling complexes to its targets, including *LFY* and *FILAMENTOUS FLOWER*. This enhances chromatin accessibility at these loci and activates transcription linked with increased H3K9ac (Wu *et al.*, 2015).

## Stochasticity in cell fate determination

It is tempting to consider cell type specification as a fully determined process because of the highly reproducible tissue growth and organogenesis. However, the cellular and molecular behaviors underlying cell type specification are often stochastic. Scientists started to realize that stochasticity is needed to create small differences between identical cells, which are then amplified and stabilized by feedback loops to begin cell differentiation (Meyer and Roeder, 2014). Experimental confirmation of this theory is the study of the variable defects in the *LEC2* mutant embryo, where *FUS3* expression appears in randomly positioned patches. The explanation might be that residual *ABSCISIC ACID INSENSITIVE3* (*ABI3*) expression fails to induce the expression of *FUS3* in some parts of the embryo while it succeeds in triggering *FUS3* expression in other parts of the embryo, and the positive feedback loop can stabilize the expression of these two genes in these embryo parts (To *et al.*, 2006).

Stochasticity happens at both cellular and molecular levels. For instance, during the growth of microtubule arrays, stochastic disassembly of individual microtubules allows them to go through various configurations and form optimal ones (Holy and Leibler, 1994; Allard *et al.*, 2010; Eren *et al.*, 2010). Studies have shown that the growth rates of leaf epidermal cells in Arabidopsis differ by several fold from each other, and change in time. This spatiotemporal variability is not related to the size of either the cell or the nucleus (Elsner *et al.*, 2012).

Stochastic gene expression has also been described in various organisms, including plants (Elowitz *et al.*, 2002; Paré *et al.*, 2009; Dar *et al.*, 2012; Ietswaart *et al.*, 2017). Gene expression noise can be divided into two types: extrinsic noise which is due to fluctuations in the cellular or external environment that affect the overall expression in a cell, and intrinsic noise which is due to the inherent fluctuations of transcription and translation of a particular gene within a cell (Elowitz *et al.*, 2002). The use of a dual reporter system in plants helped distinguish between extrinsic and intrinsic noise in Arabidopsis, and revealed that fluctuation in gene expression is coupled in neighboring cells in young leaves (Araújo *et al.*, 2017). The trichome distribution pattern also emerges stochastically, and the variability in the trichome distribution pattern correlated with stochastic cell to cell variation in *GL3* expression (Okamoto *et al.*, 2020).

One of the most compelling examples of stochasticity is from cell type specification in the sepal epidermis. Sepals have both giant cells that are very long, usually stretching one-fifth the length of the sepal, and small cells that are much smaller in size (Figure 1; Roeder *et al.*, 2010, 2012). The correct proportion of giant cells and small cells is required for the curvature of the sepal; with an altered proportion of giant cells, sepals are unable to enclose and protect the developing floral organs in the inner whorls (Roeder *et al.*, 2010, 2012). In the early stage of sepal development, the levels of the epidermis regulator *ARABIDOPSIS THALIANA MERISTEM LAYER1* (*ATML1*) fluctuate in sepal cells. When *ATML1* reaches a high level, specifically at the time of cell division, that cell will be determined to become a giant cell, whereas if the level of *ATML1* is low at this point in time, the cell will keep dividing and remain small. Thus, the stochastic fluctuations in the concentration of the TF ATML1 initiate the pattern of giant and small cells in the Arabidopsis sepal (Meyer *et al.*, 2017). The examples presented above indicate that plants utilize stochastic mechanisms to establish robust and reproducible morphology.

## How does single-cell omics contribute to understanding cell identity?

### Single-cell omics technologies

Despite the limitations of the cell type concept, classifying cells can help to understand how cells or tissues function and interact, and to reveal specific mechanisms that govern processes that may influence a plant’s growth, development, and reproduction. Recent advances in profiling molecular features at single-cell resolution provide novel insights into the understanding of cell types (see, for example, Özel *et al.*, 2021). Benefiting from the development of single-cell omics technologies, researchers can now study cellular heterogeneity at the levels of the transcriptome, epigenome, or proteome. Single-cell RNA-seq (scRNA-seq) (Tang *et al.*, 2009), ATAC-seq (Buenrostro *et al.*, 2015), ChIP-seq (Rotem *et al.*, 2015), DNA methylation (Hui *et al.*, 2018; Lee and Smallwood, 2018), metabolomics (Minakshi *et al.*, 2019), and proteomics (Marx, 2019), have emerged and are used in animal and plant research, but scRNA-seq is still the most commonly used technique. For example, scRNA-seq permits analysis of the expression profiles of thousands of individual cells at the same time and can reveal the heterogeneity within a group of cells.

An scRNA-seq experimental workflow usually begins with the dissociation and isolation of single cells from a tissue. However, in plants, the process of isolating single cells embedded in a rigid cell wall matrix is technically challenging, and it is usually achieved by incubating plant tissues with cell wall-digesting enzymes to release protoplasts. Protoplast response genes can cause artifacts in the downstream data analysis (Tucker *et al.*, 2018; Denyer *et al.*, 2019; Jean-Baptiste *et al.*, 2019; Ryu *et al.*, 2019; Shulse *et al.*, 2019), and the time and harshness of the digestion that are required to digest the cell walls differ between tissues and organs. Thus, this method is not applicable to all tissues, and longer digestion times may aggregate artifacts. An alternative way to address this issue is to isolate nuclei, for example by tissue chopping or grinding and by cell membrane lysis (e.g. [Bibr CIT0006a][Bibr CIT0039]; Kaufmann *et al.*, 2010a). It has been shown that the composition of the RNA pool from plant nuclei is representative of that from the whole cell (Deal and Henikoff, 2010). However, the isolation procedure and the loss of mechanical connection with other cell components, particularly the cytoskeleton, may change the shape of the nucleus and impact gene expression (Goswami *et al.*, 2020a). Additionally, dealing with the sparse RNA from nuclei is challenging for both the experimental and the computational parts of the work. Further optimizing tissue dissociation methods, especially for recalcitrant plant tissues is fundamentally critical to apply scRNA-seq to plant science.

What we learned from scRNA-seq is that no two cells are transcriptionally the same (Choi and Kim, 2019). Nevertheless, clustering of cells with similar expression or epigenetic profiles is often used to annotate cell types. Subclusters can reflect the variation of expression patterns among cells of the same tissue type, and may represent cell types. Taking data from roots as an example, in the stele cell cluster, there are protoxylem, phloem-like, meristematic xylem, and pericycle cells (Shulse *et al.*, 2019). However, heterogeneity may also reflect differences in cellular states, or stochastic fluctuations in gene activity (Trapnell, 2015; Wimmers *et al.*, 2018).

Cells of different ontogenetic origins may have similar functions or ‘behaviors’ in terms of gene activity. In scRNA-seq clustering, cells are grouped based on the similarity of their transcriptome. For example, the lateral root cap (LRC) cells were found to cluster with the non-hair cells and columella cells (Shulse *et al.*, 2019), which indicates that although they are different types of cells that originate from different initial cells surrounding the quiescent center (QC), they share a similar transcriptome that may provide them with the ability to protect the roots (Petricka *et al.*, 2012). It has also been shown that meristematic cells cluster together independently of precise origin. The meristematic cell clusters are close to each other and consist of meristematic cells of different identities, such as cortex identity and trichoblast identity (Denyer *et al.*, 2019). The reason for this may be because these cells share meristematic features such as a high division rate, although they have different ultimate cell fates.

The annotation of clusters in single-cell datasets other than roots—or in species other than Arabidopsis—is typically limited by the availability of tissue-specific reference datasets and specific marker genes. Such pre-knowledge of tissue-specific data can strongly enhance our capacity to annotate cells in single-cell omics datasets. This is also the case for the data from developing flowers, where at least some stage-specific and floral domain-specific datasets are available (e.g. Pajoro *et al.*, 2014; Jiao and Meyerowitz, 2010). To follow the cellular differentiation in depth, single-cell omics on fluorescence-activated cell sorting (FACS)-selected populations of green fluorescent protein (GFP)-labeled cells can be used to increase sensitivity, for example in the analysis of specific tissue types (e.g. epidermis) or cells of a certain status of differentiation (e.g. floral stem cells).

Different computational approaches try to order the transcriptome of the cells obtained by single-cell omics in some type of differentiation trajectory. The earlier methods were based on ordering cells in a pseudotime defined by similarity, for example as implemented in Monocle or Palantir (Trapnell *et al.*, 2014; Setty *et al.*, 2019). In this way, cells with similar transcriptomes were ordered together in a computationally generated pseudotime. The main problem with these methods is the assumption that transcriptome similarity is related to a similar position in the differentiation pathway, because, as we stated before, cells even from different origins can have similar transcriptomes. New approaches to infer lineage decisions are based on estimating the dynamic ratios of spliced and unspliced transcripts, for example as utilized in *velo* or *scvelo* (La Manno *et al.*, 2018; Bergen *et al.*, 2020). We can infer reaction rates of transcription, splicing, and degradation by modeling the abundance of spliced and unspliced transcripts, therefore providing an estimation of the latent time behind these dynamics.

### Towards a virtual flower: understanding cell identity in its positional context

Single-cell omics procedures are associated with the loss of positional information of plant cells. However, as discussed in the previous sections, positional information is vital for morphogenesis and cell identity in plants. By combining high-resolution imaging of marker gene activity with single-cell omics, the position of cells in their original tissue context can be predicted (Satija *et al.*, 2015; Halpern *et al.*, 2017; Cang and Nie, 2020). It is also possible to map scATAC-seq data to spatial maps of gene activity (Bravo González-Blas *et al.*, 2020). A computational framework called novoSpaRc was developed to this aim, which, in theory, can be used to *de novo* reconstruct single-cell spatial gene expression without prior spatial information, although the use of prior spatial information enhances its performance (Nitzan *et al.*, 2019). Attempts to map expression of selected regulatory genes to a virtual 3D floral meristem based on reporter gene expression and *in situ* hybridization provide a resource for this kind of computational technologies (Refahi *et al.*, 2021), and could be expanded to comprehensively cover all tissue types in the developing flower.

Although these computational tools can regain the positional information of dissociated cells, the dissociation procedure itself may cause plant cells to alter their identity, as already discussed above. So, many efforts have been made to retain tissue spacial context by using fluorescence *in situ* hybridization (FISH)-based methods, such as multiplexed error-robust FISH (MERFISH), spatially resolved transcript amplicon readout mapping (STARmap), and sequential fluorescence *in situ* hybridization (seqFISH+) (Shah *et al.*, 2016; Wang *et al.*, 2018; Peng *et al.*, 2019). SeqFISH+ can image mRNAs from up to 10 000 genes in single cells with high resolution, allowing identification of cell types based on both transcriptional profile and their spatial organization *in situ* (Peng *et al.*, 2019). Besides, seqFISH+ can also reveal subcellular mRNA localization in single cells. However, the drawback is that these kinds of methods only allow targeted studies and lack unbiased examination of the whole transcriptome. A recently published technology, expansion sequencing (ExSeq), combined expansion microscopy with long-read *in situ* RNA sequencing, resulting in a nanoscale visualization of the position of transcripts in intact tissues (Alon *et al.*, 2021). ExSeq does not need target genes, so it is unbiased compared with other *in situ* sequencing methods, as mentioned above (Alon *et al.*, 2021).

Another experimental approach to retrieve cell positional information is spatial RNA-seq. Researchers have generated high-quality RNA-seq data with maintained two-dimensional positional information by lysing histological sections on arrayed reverse transcription primers with unique positional barcodes (Ståhl *et al.*, 2016). A similar method, Slide-seq, transfers RNA from tissue sections onto DNA-barcoded drop-seq beads arrayed on a surface with known positions, allowing whole-genome sequencing of RNA with inferred locations (Rodriques *et al.*, 2019). However, these technologies can only capture tissues in a thin section, and each bead is not strictly capturing RNA from a single isolated cell. Combined with scRNA-seq, these approaches may help to map or assign single-cell transcriptomics data back into a tissue context, overcoming the need for targeted spatial expression analyses of marker genes.

In sum, parallel imaging of the expression of multiple regulatory genes or spatial omics approaches present promising avenues for mapping the expression and regulatory programs of each individual cell in a developing flower, thus taking into account position and lineage. To trace plant cell lineages, it would be interesting to test the applicability of CRISPR/Cas9 [clustered regularly interspaced palindromic repeats /CRISPR-associated protein 9]-based lineage tracing in plants (Spanjaard *et al.*, 2018).

### The promises of single-cell omics

Single-cell level transcriptomics can define cell types and identify marker genes (Trapnell, 2015). Although the clustering of cells based on ‘similarity’ has limitations (explained in the next section) in cell type identification, the techniques provide us with new insights into cellular heterogeneity: (i) single-cell technology enables the discovery of novel cell types; (ii) detects subtypes or cell states in a single cell type; and (iii) orders cells in ‘time’ along a trajectory makes it possible to infer, or at least predict, differentiation pathways (Shekhar and Menon, 2019; Torii *et al.*, 2019; Rich-Griffin *et al.*, 2020).

Single-cell omics can also provide new ways to study the function of positional signals in cell identity determination or tissue patterning. For example, a combination of single-cell omics and genetic perturbation would allow us to decipher cell type-specific auxin response pathways and dosage-dependent mechanisms in the flower. Another interesting application of scRNA-seq is to detect gradients of regulatory molecules within tissues. For example, scRNA-seq with roots has shown that some genes, such as SCARECROW and UPBEAT, represent concentration gradients along the clusters (Ryu *et al.*, 2019). Underlying mechanisms, such as mobility or gradients of regulation, will require combination of different experimental set-ups.

Since every cell of a plant is exposed to an environment that is inherently heterogeneic, single-cell omics may also help us understand cell type-specific environmental responses in the future.

### Current limitations of single-cell omics in exploring cell identities

Although single-cell omics techniques are gaining more and more popularity, we have to be aware of their limitations. For example, the power of scRNA-seq is limited because of its inability to sensitively capture all transcripts, leaving false-negative ‘zeros’ in gene expression (Dal Molin and Di Camillo, 2019). In addition, clustering methods are typically based on the assumption that cells with similar transcriptional features are ontogenetically closely related. However, the actual relationships among the profiled cells are not known because expression only represents one layer of cellular regulation, and transcriptomic similarity may not always reflect ontogenetic origin. Integrated profiling with other molecular features and spatial reconstruction will overcome these limitations (see e.g. Macaulay *et al.*, 2015; Angermueller *et al.*, 2016; Cao *et al.*, 2018).

One of the uses of scRNA-seq is to define subtypes or states within a cell type. However, fluctuations in gene expression can be caused by oscillatory cell behavior, linked to cell division, apoptosis, the circadian clock, and stochastic or bursty transcription (Stegle *et al.*, 2015; Dal Molin and Di Camillo, 2019). Nevertheless, it is possible to correct the expression noise with computational methods if these cell behaviors are not of interest (Leng *et al.*, 2015; Barron and Li, 2016).

## Conclusion and outlook

The difficulty of defining cell type conceptionally reflects the complex and dynamic nature of cells in plants. Researchers over the past decades have proposed genetic mechanisms and models to elucidate how plants build up their bodies with diverse cell types. Both the position and ‘history’ of a plant cell are important for its identity. Positional signals such as auxin, small peptides, miRNAs, and mobile TFs, as well as mechanical forces, have been shown to contribute to cell type specification and patterning in plants. TFs, including pioneer TFs associated with epigenetic modifications that evoke or consolidate cellular ‘history’, reflect the role of cell lineage in plant cell fate determination. It is the synergistic action of position- and lineage-based cell fate controlling factors, together with unregulated factors (stochasticity) that eventually determines cell identity.

Single-cell technology brings new insight into the understanding of cell identity by its advantage of studying gene expression, chromatin status, and other cellular features at the single-cell level. Despite the room for improvement, it allows us to dissect cellular heterogeneity by defining novel cell types or states that may have been neglected by classical studies. Single-cell technology can also order cells along a trajectory and make it possible to infer the origins and consequences of differentiation.

The combination of genetic analyses with single-cell technologies, reporter gene analyses, and spatial omics can be expected to deepen our knowledge on mechanisms and consequences of cell identity specification and organ patterning in plants in the future.
